# Prognostic value of procalcitonin in respiratory tract infections across clinical settings

**DOI:** 10.1186/s13054-015-0792-1

**Published:** 2015-03-06

**Authors:** Alexander Kutz, Matthias Briel, Mirjam Christ-Crain, Daiana Stolz, Lila Bouadma, Michel Wolff, Kristina B Kristoffersen, Long Wei, Olaf Burkhardt, Tobias Welte, Stefan Schroeder, Vandack Nobre, Michael Tamm, Neera Bhatnagar, Heiner C Bucher, Charles-Edouard Luyt, Jean Chastre, Florence Tubach, Beat Mueller, Philipp Schuetz

**Affiliations:** University Department of Medicine, Kantonsspital Aarau, Tellstrasse, 5001 Aarau, Switzerland; Basel Institute for Clinical Epidemiology and Biostatistics, University Hospital Basel, Hebelstrasse 10, Basel, 4031 Switzerland; Division of Endocrinology, Diabetology and Clinical Nutrition, University Hospital Basel, Hebelstrasse 10, Basel, 4031 Switzerland; Clinic of Pneumology and Pulmonary Cell Research, University Hospital Basel, Hebelstrasse 10, Basel, 4031 Switzerland; Service de Réanimation Médicale, Université Paris 7–Denis Diderot, Hôpital Bichat–Claude-Bernard, Assistance Publique–Hôpitaux de Paris (AP–HP), Henri Huchard Paris Cedex 18, Paris, 75877 France; Department of Infectious Diseases, Aarhus University Hospital, Skejby, Palle Juul-Jensens Boulevard 99, 8200 Aarhus N, Denmark; Department of Internal and Geriatric Medicine, Shanghai Jiao Tong University Affiliated Sixth People’s Hospital, Huan Hu Xi San Road, Pudong New Area, Shanghai, 201306 China; Department of Pulmonary Medicine, Hannover Medical School, Carl-Neuberg-Str. 1, Hannover, 30659 Germany; Department of Anesthesiology and Intensive Care Medicine, Düren Hospital, Roonstraße 30, Düren, 52351 Germany; Intensive Care, Universidade Federal de Minas Gerais, 6627 - Pampulha, Belo Horizonte - MG, 31270-901 Brazil; Department of Clinical Epidemiology and Biostatistics, McMaster University, 1280 Main Street West, Hamilton, Ontario L8S4L8 Canada; Service de Réanimation Médicale, Université Paris 6–Pierre et Marie Curie, Hôpital Pitié–Salpêtrière, AP-HP, 47-83 boulevard de l’Hôpital, Paris, 75651 France; AP-HP, Hôpitaux Universitaires Paris Nord Val de Seine, Département d’Epidémiologie Biostatistique et Recherche Clinique, Université Paris Diderot, Sorbonne Paris Cité, UMR 738, INSERM, UMR 738, INSERM, CIE801, 5 Rue Thomas Mann, Paris, Cedex 13 75013 France

## Abstract

**Introduction:**

Whether the inflammatory biomarker procalcitonin provides prognostic information across clinical settings and different acute respiratory tract infections (ARIs) is poorly understood. In the present study, we investigated the prognostic value of admission procalcitonin levels to predict adverse clinical outcome in a large ARI population.

**Methods:**

We analysed data from 14 trials and 4,211 ARI patients to study associations of admission procalcitonin levels and setting specific treatment failure and mortality alone at 30 days. We used multivariable hierarchical logistic regression and conducted sensitivity analyses stratified by clinical settings and ARI diagnoses to assess the results’ consistency.

**Results:**

Overall, 864 patients (20.5%) experienced treatment failure and 252 (6.0%) died. The ability of procalcitonin to differentiate patients with from those without treatment failure was highest in the emergency department setting (treatment failure area under the curve (AUC): 0.64 (95% confidence interval (CI): 0.61, 0.67), adjusted odds ratio (OR): 1.85 (95% CI: 1.61, 2.12), *P* <0.001; and mortality AUC: 0.67 (95% CI: 0.63, 0.71), adjusted OR: 1.82 (95% CI: 1.45, 2.29), *P* <0.001). In lower respiratory tract infections, procalcitonin was a good predictor of identifying patients at risk for mortality (AUC: 0.71 (95% CI: 0.68, 0.74), adjusted OR: 2.13 (95% CI: 1.82, 2.49), *P* <0.001). In primary care and intensive care unit patients, no significant association of initial procalcitonin levels and outcome was found.

**Conclusions:**

Admission procalcitonin levels are associated with setting specific treatment failure and provide the most prognostic information regarding ARI in the emergency department setting.

## Introduction

The assessment of disease severity and prediction of adverse outcome in patients with acute respiratory tract infections (ARIs) is essential to improve patient management, including therapeutic and diagnostic steps and site-of-care decisions [1-3]. For this purpose, different blood biomarkers have been evaluated to predict mortality in smaller patient cohorts and critically ill patients, but results are still somewhat controversial in regard to type of biomarker for specific ARI subpopulation and optimal cutoff levels for clinical routine [4-10].

Procalcitonin (PCT), a precursor protein of calcitonin, is currently one of the most frequently used infectious disease biomarkers in clinical practice [11]. PCT levels mirror severity and evolution of infection and are thought to be associated with poor prognosis in patients with sepsis and ARI [12]. Changes in PCT level in response to therapeutic treatment have also been reported, which suggests prognostic significance in a variety of clinical settings [13,14]. However, there is a lack of clinical data comparing PCT in different clinical settings (primary care, emergency department (ED), intensive care unit (ICU)) and across ARI diagnoses to aid in better understanding its (prognostic) value in daily clinical practice.

In the present study, we investigated the prognostic value of admission PCT levels to predict treatment failure and mortality alone in a large ARI patient population across different settings, ARI diagnoses and countries by performing an individual patient data meta-analysis.

## Material and methods

### Patients and setting

This analysis includes all patients from a previous individual patient data meta-analysis [15]. The initial meta-analysis was prespecified in collaboration with the Cochrane Database of Systematic Reviews [16]. In brief, the aim of the meta-analysis was to assess the safety and efficacy of using PCT to guide initiation and duration of antibiotic treatment in patients with ARI assigned to routine PCT measurement or standard of care without PCT measurement. This approach was used over a large range of patients with varying severities of disease in different clinical settings. Patients with a clinical diagnosis of either upper or lower ARI derived from 14 randomised or quasi-randomised trials were included. Trials focused exclusively on paediatric patients or on a purpose other than initiation and duration of antibiotic therapy were not included. Further details about identifying suitable trials were published previously [16]. No ethical approval was needed for this meta-analysis. Written informed consent was obtained from all participants within the initial trials, including consent to participate in further analyses.

### Search for and identification of trials

The initial search was prespecified and published previously [16]. In brief, suitable trials were identified by a formal search of the Cochrane Controlled Trials Registry (CCTR), MEDLINE and Embase (all from their inception to May 2011), as well as reference lists of reports describing such trials. In addition, conference proceedings (from 2006 to 2011) of the Interscience Conference on Antimicrobial Agents and Chemotherapy, the European Congress of Clinical Microbiology and Infectious Disease, the American Thoracic Society, the American Association of Respiratory Care and the American College of Chest Physicians were hand-searched. Trial registries were checked and experts contacted for further eligible trials. Two independent reviewers assessed trial eligibility on basis of titles, abstracts, full-text reports and further information obtained from investigators as needed. Further, the protocol, case report forms and unedited databases from investigators of all eligible trials were requested. Data from each trial were first checked against reported results, and queries were resolved with the principal investigator, trial data manager or statistician.

### Aims and endpoints

The aim of the present analysis was to study associations of admission PCT levels and adverse clinical outcomes. In line with the initial Cochrane meta-analysis protocol [16], the predefined primary endpoints were defined as all-cause mortality and setting specific treatment failure at 30 days. For the primary care setting, treatment failure was defined as occurrence of at least one of the following events: death, hospitalisation, ARI-specific complications (for example, empyema for lower ARI, meningitis for upper ARI), recurrent or worsening infection, and patients reporting any symptoms of an ongoing respiratory infection (for example, fever, cough, dyspnoea) at 30-day follow-up. For the ED setting, treatment failure was defined as death, ICU admission, rehospitalisation after index hospital discharge, ARI-associated complications (for example, empyema or acute respiratory distress syndrome for lower ARI), or recurrent or worsening infection within 30 days of follow-up. For the ICU setting, treatment failure was defined as death within 30 days of follow-up.

In regard to PCT as a baseline predictor, we used initial PCT levels corresponding to the PCT level drawn at the primary care visit (primary care), ED admission (ED trials) and ICU admission (ICU trials). In all trials, PCT was measured using a rapid, sensitive assay with a functional assay sensitivity of 0.06 μg/L (KRYPTOR PCT; B∙R∙A∙H∙M∙S, Hennigsdorf, Germany) and an assay time of less than 20 minutes. We used different *a priori* defined PCT cutoffs (0.1 μg/L, 0.25 μg/L, 0.5 μg/L, 2.0 μg/L) that correspond to cutoffs used in previous antibiotic stewardship trials and also in practice guidelines on the use of PCT.

### Statistical analysis

We used descriptive statistics, including mean with standard deviation, median with interquartile range (IQR) and frequencies, to describe the populations, as appropriate.

Patients were divided in two groups depending on having experienced a treatment failure or not. Because the rate of loss to follow-up in the different trials was low (<10%), we assumed that patients lost to follow-up did not undergo an event. This assumption was also verified in a time-to-event analysis where we used censoring for patients lost to follow-up.

For the primary endpoint of setting- and diagnosis-specific treatment failure or all-cause mortality alone, we calculated adjusted odds ratios (ORs) (with age and sex as additional fixed effects) and 95% confidence intervals (CIs) using multivariable hierarchical logistic regression. Discrimination of PCT levels for treatment failure or mortality was investigated by area under the receiver operating characteristic (ROC) curve and 95% CIs. For the different clinical settings (primary care, ED, ICU) and most prevalent ARI subgroups (that is, acute bronchitis, exacerbated chronic obstructive pulmonary disease (ECOPD), community acquired pneumonia (CAP)), we calculated sensitivity and specificity analyses. We used different clinically established PCT cutoffs (0.1 μg/L, 0.25 μg/L, 0.5 μg/L, 2.0 μg/L) to estimate risk prediction. If subgroups were too small, values were labelled as ‘not applicable’. For graphical display, we calculated Kaplan-Meier curves for time to death and time to adverse outcome.

Tests were carried out at 5% significance levels. Analyses were performed with STATA 12.1 software (StataCorp, College Station, TX, USA).

## Results

### Population

The study sample comprised all 4,211 intention-to-treat patients (median age: 63 years, 54.2% male) with a final diagnosis of ARI. Regarding the clinical setting, a total of 1,008 (23.9%) patients were from primary care, 2,605 (61.9%) from the ED and 598 (14.2%) from the ICU. Overall, 864 patients (20.5%) had a treatment failure and 252 (6.0%) patients died. Baseline characteristics of the overall population and stratified based on the occurrence of a treatment failure are summarized in Table 1.

**Table 1:** **Baseline characteristics** ^**a**^

**Parameter**	**All (n =4,211)**	**Patients with treatment failure (n =864)**	**Patients without treatment failure (n =3,347)**
Age (yr), median (IQR)	63 (44 to 76)	63 (45 to 76)	63 (44 to 76)
Men, n (%)	2,282 (54.2)	438 (50.7)	1,844 (55.1)
Clinical setting, n (%)			
Primary care	1,008 (23.9)	323 (37.4)	685 (20.5)
Emergency department	2,605 (61.9)	410 (47.5)	2,195 (65.6)
ICU	598 (14.2)	131 (15.2)	467 (14.0)
Primary diagnosis			
Total upper ARI, n (%)	549 (13.0)	185 (21.4)	364 (10.9)
Common cold	305 (7.2)	105 (12.2)	200 (6.0)
Rhinosinusitis, otitis	137 (3.3)	46 (5.3)	91 (2.7)
Pharyngitis, tonsillitis	107 (2.5)	34 (3.9)	73 (2.2)
Total lower ARI, n (%)	3,567 (84.7)	654 (75.7)	2,913 (87.0)
Community-acquired pneumonia	2,027 (48.1)	430 (49.8)	1,597 (47.7)
Hospital-acquired pneumonia	79 (1.9)	0 (0)	79 (2.4)
Ventilator-associated pneumonia	242 (5.7)	20 (2.3)	222 (6.7)
Acute bronchitis	531 (12.6)	105 (12.2)	426 (12.7)
Exacerbation of COPD	584 (13.9)	80 (9.3)	504 (15.1)
Exacerbation of asthma	30 (0.7)	5 (0.6)	25 (0.7)
Unspecified lower ARI	74 (1.8)	14 (1.6)	60 (1.8)
Other final diagnosis, n (%)	95 (2.3)	25 (2.9)	70 (2.1)
Procalcitonin overall (μg/L), mean, median (SD, IQR)	2.5, 0.2 (11.4, 0.1 to 0.8)	4.4, 0.2 (18.7, 0.1 to 1.2)	2.0, 0.2 (8.4, 0.1 to 0.8)

^a^ARI, Acute respiratory tract infection; COPD, Chronic obstructive pulmonary disease; ICU, Intensive care unit; IQR, Interquartile range; PCT, Procalcitonin; SD, Standard deviation.

### Procalcitonin levels in different settings and acute respiratory tract infections

**Figure 1:**
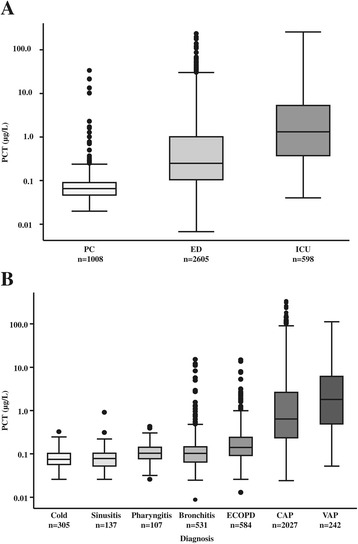
**Admission procalcitonin levels. (A)** Procalcitonin (PCT) levels stratified by setting (*P* = 0.0001). **(B)** PCT levels stratified by diagnosis (*P* = 0.0001). ECOPD, Exacerbated chronic obstructive pulmonary disease; ED, Emergency department; ICU, Intensive care unit; CAP, Community-acquired pneumonia; PC, Primary care; VAP, Ventilator-associated pneumonia.

As shown in Figure 1A, PCT levels significantly increased with higher-acuity clinical settings (that is, primary care, ED, ICU). Similar results were found when we looked at PCT levels in different types of ARI with higher levels in more severe ARIs (that is, ECOPD, CAP, ventilator-associated pneumonia) (Figure 1B).

### Association of admission procalcitonin levels and adverse outcome

**Figure 2:**
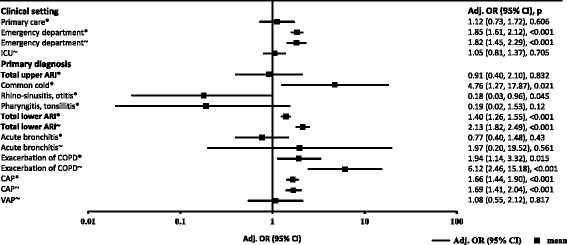
**Multivariate regression analysis for estimation of predictive value of procalcitonin levels on admission stratified by adverse events and mortality in different settings and diagnoses.** *Treatment failure; ~Mortality; Adj., Adjusted; ARI, Acute respiratory tract infection; CAP, Community-acquired pneumonia; CI, Confidence interval; ICU, Intensive care unit; COPD, Chronic obstructive pulmonary disease OR, Odds ratio; VAP, Ventilator-associated pneumonia.

Table 2 summarizes the results of logistic regression analysis as a measure of association and AUC as a measure of discrimination for both endpoints: treatment failure and mortality. PCT levels in ED patients were significantly associated with treatment failure (AUC: 0.64 (95% CI: 0.61, 0.67), adjusted OR: 1.85 (95% CI: 1.61, 2.12), *P* <0.001) and mortality (AUC: 0.67 (95% CI: 0.63, 0.71), adjusted OR: 1.82 (95% CI: 1.45, 2.29), *P* <0.001), whereas no significant effect was seen in primary care or ICU patients. In patients with common cold, PCT could significantly predict treatment failure (AUC: 0.58 (95% CI: 0.51, 0.64), adjusted OR: 4.76 (95% CI: 1.27, 17.87), *P* = 0.021). Nevertheless, PCT did not deliver supplementary prognostic information in the overall upper ARI population. In lower ARI, PCT was significantly associated with treatment failure (AUC: 0.57 (95% CI: 0.54, 0.59), adjusted OR: 1.40 (95% CI: 1.26, 1.55), *P* <0.001) and mortality (AUC: 0.71 (95% CI: 0.68, 0.74), adjusted OR: 2.13 (95% CI: 1.82, 2.49), *P* <0.001). In patients with acute bronchitis (AUC: 0.91 (95% CI: 0.88, 0.95), adjusted OR: 1.97 (95% CI: 0.20, 19.52), *P* = 0.561) and ECOPD (AUC: 0.79 (95% CI: 0.69, 0.90), adjusted OR: 6.12 (95% CI: 2.46, 15.18), *P* <0.001), admission PCT levels were good predictors to identify patients at risk for mortality. Significantly different PCT levels in patients with versus without events (treatment failure or mortality) were also observed in ECOPD and CAP, as detailed in Table 2. Figure 2 shows the results of the adjusted regression analysis for treatment failure and mortality in the different populations studied.

**Table 2:** **Procalcitonin levels at day 0 for prediction of severe adverse events in patients with acute respiratory infection** ^**a**^

	**Event**	**PCT (μg/L) in patients with treatment failure**	**PCT (μg/L) in patients without treatment failure**	**Adjusted OR (95% CI)**	**AUC (95% CI)**	***P*** **-value**
Clinical setting, mean, median (SD, IQR)						
Primary care	Treatment failure	0.26, 0.07 (2.22, 0.05 to 0.09)	0.14, 0.07 (0.83, 0.05 to 0.09)	1.12 (0.73, 1.72)	0.50 (0.46, 0.54)	0.606
Emergency department	Treatment failure	5.26, 0.51 (17.86, 0.17 to 2.71)	1.85, 0.23 (8.09, 0.10 to 0.80)	1.85 (1.61, 2.12)	0.64 (0.61, 0.67)	**<0.001**
	Mortality	5.85, 0.54 (22.53, 0.21 to 3.17)	2.22, 0.24 (9.33, 0.10 to 0.96)	1.82 (1.45, 2.29)	0.67 (0.63, 0.71)	**<0.001**
ICU	Mortality	12.06, 1.15 (35.21, 0.33 to 6.34)	6.59, 1.36 (14.07, 0.37 to 5.07)	1.05 (0.81, 1.37)	0.50 (0.44, 0.56)	0.705
Primary diagnosis						
Total upper ARI, n (%)	Treatment failure	0.07, 0.06 (0.04, 0.05 to 0.08)	0.07, 0.06 (0.05, 0.04 to 0.08)	0.91 (0.40, 2.10)	0.50 (0.45, 0.55)	0.832
Common cold	Treatment failure	0.07, 0.06 (0.03, 0.05 to 0.08)	0.06, 0.05 (0.03, 0.04 to 0.08)	4.76 (1.27, 17.87)	0.58 (0.51, 0.64)	**0.021**
Rhinosinusitis, otitis	Treatment failure	0.06, 0.05 (0.03, 0.03 to 0.08)	0.08, 0.06 (0.08, 0.04 to 0.09)	0.18 (0.03, 0.96)	0.39 (0.29, 0.50)	**0.045**
Pharyngitis, tonsillitis	Treatment failure	0.08, 0.07 (0.05, 0.06 to 0.10)	0.09, 0.08 (0.05, 0.06 to 0.11)	0.19 (0.02, 1.53)	0.42 (0.31, 0.54)	0.120
Total lower ARI, n (%)	Treatment failure	5.64, 0.38 (21.20, 0.11 to 1.91)	2.34, 0.23 (8.97, 0.09 to 1.02)	1.40 (1.26, 1.55)	0.57 (0.54, 0.59)	**<0.001**
	Mortality	9.3, 0.95 (30.25, 0.30 to 5.11)	2.49, 0.22 (9.59, 0.09 to 1.04)	2.13 (1.82, 2.49)	0.71 (0.68, 0.74)	**<0.001**
Acute bronchitis	Treatment failure	0.10, 0.07 (0.11, 0.05 to 0.11)	0.22, 0.08 (0.93, 0.05 to 0.12)	0.77 (0.40, 1.48)	0.47 (0.41, 0.53)	0.430
	Mortality	0.22, 0.22 (0.04, 0.19 to 0.24)	0.20, 0.08 (0.84, 0.05 to 0.11)	1.97 (0.20, 19.52)	0.91 (0.88, 0.95)	0.561
Exacerbation of COPD	Treatment failure	0.40, 0.14 (1.30, 0.08 to 0.27)	0.23, 0.10 (0.67, 0.07 to 0.17)	1.94 (1.14, 3.32)	0.60 (0.53, 0.67)	**0.015**
	Mortality	1.06, 0.25 (2.70, 0.18 to 0.62)	0.23, 0.10 (0.64, 0.07 to 0.18)	6.12 (2.46, 15.18)	0.79 (0.69, 0.90)	**<0.001**
Community-acquired pneumonia	Treatment failure	8.30, 1.00 (25.76, 0.28 to 5.19)	3.13, 0.41 (10.56, 0.17 to 1.57)	1.66 (1.44, 1.90)	0.61 (0.58, 0.64)	**<0.001**
	Mortality	10.66, 1.01 (32.95, 0.33 to 5.67)	3.53, 0.44 (11.63, 0.18 to 1.76)	1.69 (1.41, 2.04)	0.62 (0.58, 0.66)	**<0.001**
Ventilator-associated pneumonia	Mortality	4.27, 1.29 (6.43, 0.39 to 5.87)	5.34, 1.42 (11.81, 0.37 to 4.47)	1.08 (0.55, 2.12)	0.51 (0.38, 0.65)	0.817

^a^ARI, Acute respiratory tract infection; AUC, Area under the curve; CI, Confidence interval; COPD, Chronic obstructive pulmonary disease; ICU, Intensive care unit; IQR, interquartile range; OR, odds ratio; PCT, procalcitonin; SD, standard deviation. Statistically significant values are shown in bold.

**Figure 3:**
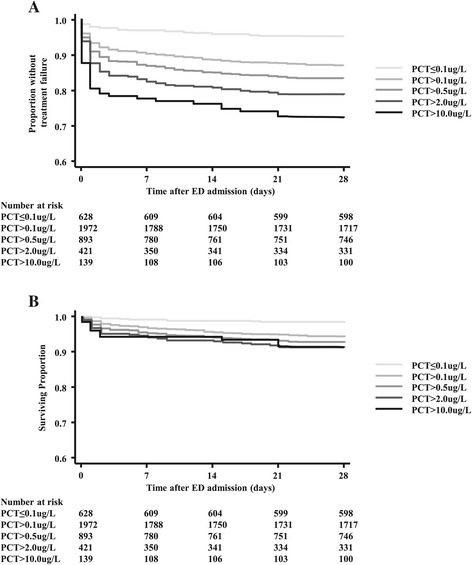
**Association between time to severe adverse events and admission procalcitonin levels in emergency department patients. (A)** Time to treatment failure is significantly shorter in emergency department (ED) patients with higher procalcitonin (PCT) levels on admission. (*P* <0.0001. **(B)** Time to death is significantly shorter in ED patients with higher PCT levels on admission (*P* <0.02). Treatment failure is defined as death, ICU admission, rehospitalisation, complications or recurrent or worsening infection within 28 days.

**Figure 4:**
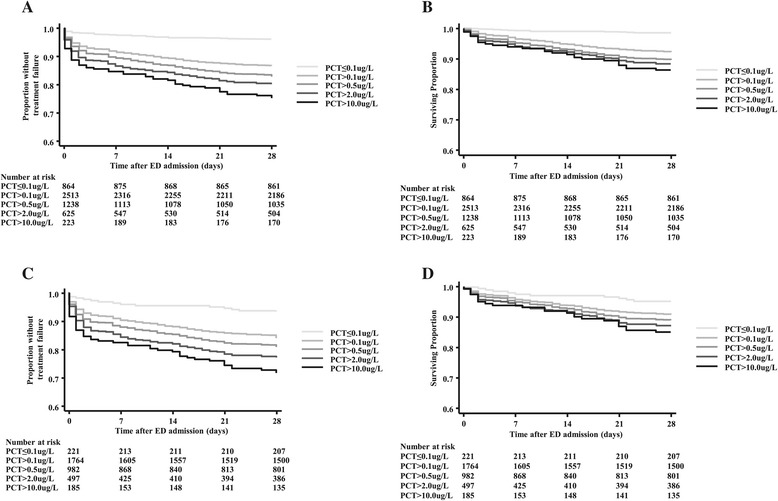
**Association between time to severe adverse events and admission procalcitonin levels in lower acute respiratory tract infection and community-acquired pneumonia patients. (A)** Time to treatment failure is significantly shorter in lower acute respiratory tract infection (ARI) patients with higher procalcitonin (PCT) levels on admission (*P* <0.0001). **(B)** Time to death is significantly shorter in lower ARI patients with higher PCT levels on admission (*P* <0.0001). **(C)** Time to treatment failure is significantly shorter in patients with community-acquired pneumonia (CAP) with higher PCT levels on admission (*P* <0.01). **(D)** Time to death is significantly shorter in patients with CAP with higher PCT levels on admission (*P* <0.01). ED, Emergency department. Treatment failure is defined as death, ICU admission, rehospitalisation, complications or recurrent or worsening infection within 28 days.

PCT in the different cutoff ranges significantly separated ED patients in regard to the time to treatment failure (Figure 3A) and time to mortality (Figure 3B) (*P* <0.0001 and *P* = 0.02, respectively). Similar analyses did not show significant results in the primary care and ICU settings (data not shown). In the overall lower ARI population, as well as in CAP patients, PCT also showed significant separation in regard to the time to treatment failure (*P* <0.0001 and *P* <0.01, respectively) and time to mortality (*P* <0.0001 and *P* <0.01, respectively) (Figure 4).

Tables 3 and 4 gives an overview of diagnostic measures (including sensitivity, specificity, negative predictive values (NPVs) and positive predictive values (PPVs)) of PCT at different PCT cutoff levels. In primary care patients, a cutoff of 0.25 μg/L had a high PPV of 45.8% (95% CI: 25.6, 67.2) and a high specificity of 98.1% (95% CI: 96.8, 99.0) for treatment failure. At the same cutoff, we found a high NPV of 89.2% (95% CI: 87.4, 90.8) and a sensitivity of 65.6% (95% CI: 60.8, 70.2) for treatment failure and a NPV of 97.5% (95% CI: 96.5, 98.3) and a sensitivity of 72.5% (95% CI: 63.6, 80.3) for mortality in ED patients. Similarly, in patients with lower ARIs, a low PCT cutoff showed high levels of sensitivity, whereas levels of specificity were higher with higher PCT cutoffs. In patients with CAP, the NPV of PCT at the 0.1 and 0.25 μg/L cutoffs were high for treatment failure (85.5% (95% CI: 80.2, 89.9) and 84.9% (95% CI: 81.9, 87.6)) as well as for mortality (95.5% (95% CI: 91.8, 97.8) and 94.2% (95% CI: 92.2, 95.9)). For ICU patients, diagnostic measures remained somewhat undetermined at all the different PCT cutoffs.

**Table 3:** **Procalcitonin cutoffs (0.1 and 0.25 μg/L) for risk prediction in different clinical settings and in various acute respiratory infection subgroups** ^**a**^

**Cutoff (μg/L)**	**0.1 μg/L**				**0.25 μg/L**			
	**Sensitivity (%)**	**Specificity (%)**	**PPV (%)**	**NPV (%)**	**Sensitivity (%)**	**Specificity (%)**	**PPV (%)**	**NPV (%)**
	**(95% CI)**	**(95% CI)**	**(95% CI)**	**(95% CI)**	**(95% CI)**	**(95% CI)**	**(95% CI)**	**(95% CI)**
PC								
Treatment failure	19.8 (15.6, 24.6)	81.3 (78.2, 84.1)	33.3 (26.7, 40.5)	68.2 (64.9, 71.4)	3.41 (1.71, 6.01)	98.1 (96.8, 99.0)	45.8 (25.6, 67.2)	68.3 (65.2, 71.2)
ED								
Treatment failure	86.1 (82.4, 89.3)	26.1 (24.2, 28.0)	17.9 (16.2, 19.7)	90.9 (88.4, 93.1)	65.6 (60.8, 70.2)	53.1 (51.0, 55.2)	20.8 (18.6, 23.1)	89.2 (87.4, 90.8)
Mortality	92.5 (86.2, 96.5)	25.0 (23.3, 26.7)	5.63 (4.65, 6.74)	98.6 (97.3, 99.3)	72.5 (63.6, 80.3)	51.2 (49.3, 53.2)	6.71 (5.4, 8.2)	97.5 (96.5, 98.3)
ICU								
Mortality	95.2 (89.8, 98.2)	4.21 (2.43, 6.75)	24.6 (20.9, 28.7)	72.7 (49.8, 89.3)	80.8 (72.8, 87.3)	18.9 (15.1, 23.3)	24.7 (20.6, 29.2)	75.0 (65.1, 83.3)
Total lower ARI								
Treatment failure	76.4 (72.9, 79.6)	28.2 (26.5, 29.9)	19.6 (18.1, 21.2)	83.9 (81.4, 86.1)	57.4 (53.5, 61.3)	52.8 (51.0, 54.7)	21.8 (19.9, 23.9)	84.4 (82.6, 86.0)
Mortality	94.5 (90.8, 97.0)	28.9 (27.4, 30.5)	8.89 (7.8, 10.1)	98.6 (97.7, 99.3)	78.1 (72.2, 83.2)	53.0 (51.3, 54.8)	10.9 (9.42, 12.4)	97.1 (96.2, 97.8)
Bronchitis								
Treatment failure	28.6 (20.2, 38.2)	68.0 (63.3, 72.4)	18.1 (12.5, 24.8)	79.4 (74.9, 83.4)	3.81 (1.05, 9.47)	92.5 (89.5, 94.8)	11.1 (3.11, 26.1)	79.6 (75.7, 83.0)
Mortality	100 (15.8, 100)	68.9 (64.8, 72.9)	1.20 (0.15, 4.28)	100 (99.0, 100)	0.00 (0.00, 84.2)	93.2 (90.7, 95.2)	0.00 (0.00, 9.74)	99.6 (98.5, 100)
ECOPD								
Treatment failure	60.0 (48.4, 70.8)	49.8 (45.3, 54.3)	16.0 (12.0, 20.6)	88.7 (84.4, 92.1)	27.5 (18.1, 38.6)	85.9 (82.5, 88.8)	23.7 (15.5, 33.6)	88.1 (84.9, 90.9)
Mortality	82.4 (56.6, 96.2)	49.4 (45.2, 53.6)	4.67 (2.57, 7.71)	98.9 (96.9, 99.8)	47.1 (23.0, 72.2)	85.0 (81.7, 87.8)	8.60 (3.79, 16.2)	98.2 (96.5, 99.2)
CAP								
Treatment failure	92.5 (89.5, 94.8)	12.1 (10.5, 13.8)	22.2 (20.3, 24.2)	85.5 (80.2, 89.9)	77.1 (72.8, 81.0)	35.0 (32.6, 37.4)	24.3 (22.1, 26.7)	84.9 (81.9, 87.6)
Mortality	94.9 (90.9, 97.5)	11.8 (10.3, 13.4)	10.6 (9.20, 12.1)	95.5 (91.8, 97.8)	81.2 (75.1, 86.4)	33.9 (31.7, 36.1)	11.9 (10.2, 13.8)	94.2 (92.2, 95.9)

^a^ARI, Acute respiratory tract infection; CAP, Community-acquired pneumonia; CI, Confidence interval; ECOPD, Exacerbated chronic obstructive pulmonary disease; ED, Emergency department; ICU, Intensive care unit; NA, Not applicable due to small sample size; NPV, Negative predictive value; PC, Primary care; PCT, Procalcitonin; PPV, Positive predictive value.

**Table 4:** **Procalcitonin cutoffs (0.5 and 2.0 μg/L) for risk prediction in different clinical settings and in various acute respiratory infection subgroups** ^**a**^

**Cutoff (μg/L)**	**0.5 μg/L**				**2.0 μg/L**			
	**Sensitivity (%)**	**Specificity (%)**	**PPV (%)**	**NPV (%)**	**Sensitivity (%)**	**Specificity (%)**	**PPV (%)**	**NPV (%)**
	**(95% CI)**	**(95% CI)**	**(95% CI)**	**(95% CI)**	**(95% CI)**	**(95% CI)**	**(95% CI)**	**(95% CI)**
PC								
Treatment failure	1.86 (0.69, 4.00)	98.7 (97.5, 99.4)	40.0 (16.3, 67.7)	68.0 (65.0, 70.9)	NA	NA	NA	NA
ED								
Treatment failure	50.2 (45.3, 55.2)	68.6 (66.6, 70.6)	23.1 (20.3, 26.0)	88.0 (86.4, 89.6)	27.6 (23.3, 32.2)	85.9 (84.4, 87.4)	26.8 (22.7, 31.3)	86.4 (84.9, 87.8)
Mortality	52.5 (43.2, 61.7)	66.5 (64.6, 68.4)	7.05 (5.46, 8.94)	96.7 (95.7, 97.5)	28.3 (20.5, 37.3)	84.4 (82.9, 85.8)	8.08 (5.66, 11.1)	96.1 (95.1, 96.8)
ICU								
Mortality	67.2 (58.2, 75.3)	30.8 (26.2, 35.7)	24.2 (19.8, 29.1)	74.1 (66.5, 80.7)	37.6 (29.1, 46.7)	58.2 (53.0, 63.2)	22.8 (17.3, 29.2)	73.9 (68.5, 78.8)
Total lower ARI								
Treatment failure	44.9 (41.0, 48.8)	66.4 (64.7, 68.2)	23.5 (21.2, 26.0)	84.0 (82.4, 85.5)	24.4 (21.1, 27.9)	83.4 (82.0, 84.8)	25.3 (21.9, 28.9)	82.8 (81.3, 84.1)
Mortality	61.2 (54.7, 67.4)	66.2 (64.5, 67.8)	11.7 (9.97, 13.6)	95.9 (95.0, 96.7)	33.8 (27.8, 40.2)	83.1 (81.8, 84.4)	12.8 (10.3, 15.7)	94.5 (93.6, 95.3)
Bronchitis								
Treatment failure	0.95 (0.02, 5.19)	96.0 (93.7, 97.7)	5.56 (0.14, 27.3)	79.7 (75.9, 83.1)	NA	NA	NA	NA
Mortality	0.00 (0.00, 84.2)	96.6 (94.7, 98.0)	0.00 (0.00, 18.5)	99.6 (98.6, 100)	NA	NA	NA	NA
ECOPD								
Treatment failure	11.3 (5.28, 20.3)	93.8 (91.3, 95.8)	22.5 (10.8, 38.5)	86.9 (83.8, 89.6)	2.50 (0.30, 8.74)	98.8 (97.4, 99.6)	25.0 (3.19, 65.1)	86.4 (83.3, 89.1)
Mortality	29.4 (10.3, 56.0)	93.8 (91.5, 95.6)	12.5 (4.19, 26.8)	97.8 (96.2, 98.9)	5.88 (0.15, 28.7)	98.8 (97.5, 99.5)	12.5 (0.32, 52.7)	97.2 (95.5, 98.4)
CAP								
Treatment failure	62.5 (57.7, 67.1)	54.1 (51.6, 56.6)	27.0 (24.2, 29.9)	84.2 (81.8, 86.4)	34.9 (30.4, 39.7)	77.7 (75.5, 79.7)	29.8 (25.8, 34.0)	81.5 (79.4, 83.4)
Mortality	64.0 (56.8, 70.7)	52.2 (49.8, 54.5)	12.8 (10.8, 15.1)	92.9 (91.2, 94.4)	36.0 (29.3, 43.2)	76.2 (74.1, 78.1)	14.3 (11.3, 17.7)	91.5 (90.0, 92.9)

^a^ARI, Acute respiratory tract infection; CAP, Community-acquired pneumonia; CI, Confidence interval; ECOPD, Exacerbated chronic obstructive pulmonary disease; ED, Emergency department; ICU, Intensive care unit; NA, Not applicable due to small sample size; NPV, Negative predictive value; PC, Primary care; PCT, Procalcitonin; PPV, Positive predictive value.

## Discussion

The findings of this analysis, which included a large population with different types and severities of ARI from previous randomised controlled trials, are threefold. First, we found an increase in PCT levels across clinical settings and ARI diagnoses, demonstrating that the current practice of using different cutoff levels in different clinical settings is mandatory. Second, initial PCT levels were significantly associated with setting-specific treatment failure and mortality in the overall population. Third, the prognostic information derived from initial PCT levels in primary care and ICU patients was rather low, whereas in the ED setting and in ECOPD and bronchitis patients, admission PCT levels provided prognostic information and thereby may improve initial risk stratification in these patient populations.

An accurate and fast assessment of disease severity and predictions regarding a patient’s clinical course potentially assist patients and physicians with setting-appropriate expectations regarding the illness. Such predictions are of particular importance to ensuring efficient use of health care and hospital resources and are indispensable for choosing optimal therapeutic options in the initial management of ARIs [17]. This includes decisions regarding site of care, diagnostic evaluation and assessment for appropriate early discharge [18].

The role of prognostication is also acknowledged by respiratory infection guidelines, which recommend stratifying patients with CAP on the basis of predicted risk for mortality using validated risk scores (that is, the Pneumonia Severity Index (PSI) or CURB-65 score) [19,20]. Clinical risk scores are somewhat limited by practicality and risk for miscalibration due to different patient populations, and therefore they have only moderate operational characteristics [21]. Also, these scores are validated only for CAP and not for other ARIs. Thus, there is interest in additional prognosticating mechanisms, throughout all clinical settings and subgroups of ARIs, using newly available biomarkers (such as PCT) that are objectively and rapidly measurable, as well as responsive to clinical recovery, and that add relevant, reliable, real-time information.

The prognostic potential of PCT in patients with respiratory infections has been evaluated in different studies, mainly in patients with CAP and sepsis. Most studies have found PCT levels to be increased in patients not surviving their disease compared with survivors, but with only moderate prognostic accuracy. In line with our results, in a large CAP cohort in the United States [22], the greatest benefit of PCT was found in patients classified as high risk on the basis of PSI score. Having a PCT <0.1 μg/L virtually excluded mortality in these high-risk patients. In a Swiss study, researchers found that initial PCT levels did not improve clinical risk scores for mortality prediction [23]. Subsequent repeated measurements of PCT in this population demonstrated improved clinical outcomes with falling PCT levels. In addition, the study found that PCT was more helpful in predicting treatment failure other than mortality, such as ICU admission or CAP-related complications. For these outcomes, PCT significantly improved clinical risk scores. In another large CAP study, done in Germany and including mostly low-risk patients, researchers found that PCT was a fair predictor of mortality and significantly improved clinical risk scores [24].

According to these studies, our findings are generally congruent. In low-risk patients (primary care) with respiratory infections, low PCT levels <0.1 μg/L correctly identified patients at lower risk for treatment failure with a high NPV. In higher-risk populations (CAP, ICU), increased levels of PCT had high PPV mainly for mortality prediction. In ED patients and in patients with lower ARIs, PCT was a good predictor of treatment failure and mortality.

Nonetheless, several limitations should be considered when interpreting our results. First, we carried out a secondary analysis of a meta-analysis. It was designed to focus on a different question, that is, whether using PCT to guide initiation and duration of antibiotic treatment in patients with ARIs in primary care, ED and ICUs is safe and efficient in a broader patient population. Second, we did not investigate the effect of repeated measurements that might add supplementary prognostic information to admission levels. Third, we focused only on interventional trials as specified in the meta-analysis protocol and did not include any observational data that would potentially allow for larger sample sizes in the different subgroups and increases in the patient spectrum, as in observational studies less rigorous exclusion criteria usually apply. Fourth, we focused on adverse outcomes as specified in the original trials, but we were not able to look into some adverse outcomes, such as septic shock and respiratory failure, among others. Fifth, clinical risk scores such as CURB-65 and PSI for CAP and Acute Physiology and Chronic Health Evaluation for ICU patients were not routinely available and thus were not included in this analysis. Similarly, we were not able to obtain more detailed baseline data on all patients, including comorbidities and other risk factors, which would have allowed a more rigorous adjustment in the statistical models. Although this study provides new and clinically relevant information about the prognostic value of initial admission PCT values in different clinical setting and for different upper and lower ARIs, it remains unclear whether an improved initial prognostic assessment based on PCT level would translate into better triage decisions and outcomes in patients. Sixth, randomised controlled outcome studies need to be conducted for patients with ARIs in the ED and ICU to investigate whether real-life measurement of PCT adds useful prognostic information and thereby improves the daily clinical management and outcomes of patients.

## Conclusions

This is the first large-scale study in which the prognostic value of admission PCT levels has been investigated throughout all clinical settings in the field of ARIs. PCT levels mainly provide prognostic information for risk stratification of patients in the ED setting and in patients with low to moderate ARIs. Future randomised controlled studies must address whether adding PCT to initial risk assessment can improve patient management and prognostication.

## Key messages

- We found an increase in PCT levels across the clinical settings and ARI diagnoses, supporting the current practice of using specific PCT cutoff levels in different clinical settings.
- PCT levels are significantly associated with treatment failure and mortality in the overall population.
- The prognostic information of initial PCT levels in primary care and ICU patients is rather low, whereas admission PCT levels provide important prognostic information in the ED setting and in patients with low to moderate ARIs.

## Abbreviations

ARI: Acute respiratory tract infection
AUC: Area under the curve
CAP: Community-acquired pneumonia
CI: Confidence interval
ECOPD: Exacerbated chronic obstructive pulmonary disease
ED: Emergency department
ICU: Intensive care unit
IQR: Interquartile range
OR: Odds ratio
PCT: Procalcitonin
PSI: Pneumonia Severity Index
ROC: Receiver operating characteristic
RTI: Respiratory tract infection
VAP: Ventilator-associated pneumonia
